# Singleton pregnancies after in vitro fertilization in Estonia: a register-based study of complications and adverse outcomes in relation to the maternal socio-demographic background

**DOI:** 10.1186/s12884-019-2194-x

**Published:** 2019-01-29

**Authors:** Kaja Rahu, Kärt Allvee, Helle Karro, Mati Rahu

**Affiliations:** 1 Department of Epidemiology and Biostatistics, National Institute for Health Development, Hiiu 42, 11619 Tallinn, Estonia; 2 Estonian Medical Birth Registry, National Institute for Health Development, Hiiu 42, 11619 Tallinn, Estonia; 3 Department of Obstetrics and Gynecology, Institute of Clinical Medicine, University of Tartu, L. Puusepa 8, 51014 Tartu, Estonia; 4 Tartu University Hospitals Womens Clinic, L. Puusepa 8, 51014 Tartu, Estonia

**Keywords:** In vitro fertilization, Pregnancy outcome, Pregnancy-related complications, Primipara, Singleton, Socio-demographic background

## Abstract

**Background:**

An increased risk of adverse conditions related to in vitro fertilization (IVF) pregnancies has been repeatedly reported. Our study aimed to summarize outcome differences between pregnancies after IVF and after spontaneous conception (SC) in Estonia.

**Methods:**

Data on all liveborn singletons to primiparas women aged 25–40 years during the period 2005–2014 were obtained from the Estonian Medical Birth Registry. There were 1778 and 33,555 newborns in the IVF and SC cohort, respectively. The relative risk of pregnancy-related complications and adverse pregnancy outcomes in the IVF cohort in comparison with the SC cohort was quantified by prevalence proportion ratios (RR) with 95% confidence intervals (CI) using modified Poisson regression models adjusted for maternal age, education, ethnicity, marital status and study period.

**Results:**

The cohort of IVF singletons experienced a higher risk of preterm birth (RR 1.51; 95% CI 1.28–1.78), iatrogenic preterm birth (RR 1.62; 95% CI 1.32–1.98), very preterm birth (RR 1.49; 95% CI 1.00–2.23), low birthweight (RR 1.47; 95% CI 1.20–1.80), congenital anomalies (RR 1.51; 95% CI 1.08–2.11), and admission to a neonatal intensive care unit (RR 1.13; 95% CI 1.01–1.26). Somewhat elevated risk of spontaneous preterm birth did not reach statistical significance (RR 1.32; 95% CI 0.97–1.80). IVF mothers were at increased risk of placenta previa (RR 7.15; 95% CI 4.04–12.66), placental abruption (RR 2.12; 1.43–3.14) and cesarean section (RR 1.28; 95% CI 1.20–1.37). The risk of pre-eclampsia was borderline (RR 1.25; 95% CI 0.98–1.59). Adjustment for maternal age attenuated the associations between IVF and adverse outcomes. Maternal education, ethnicity and marital status had no effect on the magnitude of the risk estimates.

**Conclusions:**

The increased risk of pregnancy-related complications and adverse pregnancy outcomes was observed in the Estonian cohort of IVF singletons in comparison with the cohort of SC singletons. The relative risk estimates grew with maternal age but were not influenced by the maternal education, ethnicity and marital status. To monitor the efficacy and safety of the used assisted reproductive technology, a specialized country-wide register should be created in Estonia.

## Background

In vitro fertilization (IVF) as an effective infertility treatment procedure was introduced in Estonia in 1997. According to the Artificial Insemination and Embryo Protection Act, women aged 18–50 years and with active legal capacity can undergo artificial insemination by transferring maximally three embryos per cycle [1]. Currently, there are five IVF treatment clinics, all of which have a contract with the Estonian Health Insurance Fund (EHIF) that administers the compulsory solidarity-based health insurance system [2]. EHIF covers an unlimited number of treatment cycles for insured women up to 40 years of age. Women aged 41–50 years must pay for the IVF treatment themselves.

By the Estonian Medical Birth Registry (EMBR) data, 1.2% of newborns in 2005 were conceived by IVF, including 0.7% of singletons and 19.4% of multiples. The respective figures in 2014 were 3.5, 2.6 and 32.9%. The proportion of multiple births among births after IVF declined from 26.1% in 2005 to 16.9% in 2014. One embryo was transferred in 32.5%, two embryos in 58.5% and three embryos in 8.9% of cycles in 2014 [3].

The health of IVF children and their mothers has been monitored since the first IVF baby was born in 1978 [4]. The major risk of adverse outcomes among IVF newborns is attributed to the higher proportion of multiple births among them [5–7]. However, an increased risk of complications and adverse outcomes in singleton pregnancies after IVF compared with spontaneously conceived (SC) singleton pregnancies has been repeatedly summarized in meta-analyses [8–11]. In the studies with detailed data about IVF procedures, it has been shown that even singletons born after a single embryo transfer have somewhat poorer perinatal outcome than SC singletons [12, 13]; however, a recent study concluded no increased risks [14]. Even though there is evidence that embryo freezing leads to better pregnancy outcome, this method has still resulted in elevated risks [15–17]. The causes of poorer outcome in pregnancies after IVF have been found to be due to both the IVF treatment itself and the underlying infertility [10, 18, 19]. Conversely, Seggers et al. [20] concluded that IVF treatment did not have an independent adverse effect on the birthweight.

Previous studies have shown that IVF pregnancies are more common among women aged ≥35 years, married or cohabiting, or with higher socioeconomic status [14, 15, 20, 21]. However, Qin et al. [19] reported a higher proportion of IVF pregnancies among less educated women. There is some evidence that mothers’ socioeconomic status has no effect on the relative risk of adverse outcomes in pregnancies after IVF [21].

Given the higher risk of PTB and lower mean birthweight among SC singletons born to unmarried, non-Estonian or less educated mothers in Estonia [22], and relying exclusively on the EMBR data, we focused on the differences in complications and adverse outcomes between singleton pregnancies after IVF and after SC in relation to the maternal socio-demographic background.

## Methods

We used data from the EMBR, which collects information on maternal socio-demographic background, reproductive history, pregnancy risk factors, pregnancy and delivery complications, and on perinatal outcomes for each birth (≥22 weeks of gestation) with unique personal identification numbers for mothers and newborns [22, 23]. IVF status is registered as yes/no and is defined as a birth after embryo transfer following conventional in vitro insemination or intracytoplasmic sperm injection [24]. Data quality in the EMBR is estimated to be acceptable [25]. We identified women who were residents of Estonia and delivered at age of 25–40 years. The study was restricted to the period of 2005–2014 as for infants born in these years and with maternal age of ≤40 years, the IVF status in the EMBR has been previously validated through the record linkage with the databases of the EHIF and the infertility treatment clinics [23]. After the unrecorded IVF status was corrected in the birth records, 518 newborns moved from the SC cohort to the IVF cohort in the studied sample. To reduce confounding, a more homogenous study sample was created to include only liveborn singletons to primiparas; thus, six stillbirths after IVF (0.3%) and 109 after SC (0.3%) pregnancies were excluded. In total, the IVF cohort of 1778 newborns and a comparison cohort of 33,559 SC newborns were eligible. As the EMBR does not collect information on ovulation induction or intrauterine insemination, the SC cohort could include the singletons born after those treatments.

The study period was split into two sub-periods: 2005–2009 and 2010–2014. The maternal socio-demographic background was described using the following characteristics: age, stratified into three age groups of 25–29, 30–34 and 35–40 years; education, categorized as higher (university or professional higher), secondary (secondary or secondary specialized) and basic (9 years or less); ethnicity, categorized as Estonian, Russian and other; and marital status, categorized as married, cohabiting and single (single, divorced or widowed). Births with unknown maternal socio-demographic characteristics were excluded before the analysis (four records with missing maternal educational level in the SC cohort).

The adverse pregnancy outcomes of interest were selected as preterm birth (PTB; < 37 weeks), very preterm birth (VPTB; < 32 weeks), low birthweight (LBW; < 2500 g), very low birthweight (VLBW; < 1500 g), small for gestational age (SGA; defined as birthweight more than 2 standard deviations below the population sex- and parity-specific mean by completed gestational week), low Apgar score at 5 min (< 7), congenital anomalies (ICD-10 Q00–Q99, diagnosed in the first week of life), and admission to a neonatal intensive care unit (NICU). PTB was separated to iatrogenic PTB, defined as birth after induction of labor or pre-labor cesarean section, and to spontaneous PTB otherwise. Maternal pregnancy-related complications included pre-eclampsia (O11, O14), placenta previa (O44), placental abruption (O45), and cesarean section (planned or emergency). Occurrence of congenital anomalies, admission to a NICU, pre-eclampsia, placenta previa and placental abruption were marked in the birth notification form using tick boxes, where checked meant “yes” and not checked meant “no” or “unknown”. Gestational age was determined by the best clinical estimate (ultrasound before the 21st week, 97.7 and 97.1% in the IVF cohort and SC cohort, respectively). Deaths in the IVF and SC cohort were obtained via linkage with the national causes of death registry. The number of early neonatal deaths (two cases), late neonatal deaths (one case) and infant deaths after the neonatal period (none) in the IVF cohort was too small for quantitative analysis.

The proportion of the IVF births in each category of maternal background characteristics and pregnancy outcomes was calculated. The relative risks of pregnancy-related complications and adverse pregnancy outcomes in the IVF cohort in comparison with the SC cohort was quantified by prevalence proportion ratios (RR) using modified Poisson regression models with a robust error variance [26]. In addition to the single outcomes, combined categories of pregnancy-related complications (at least one of the following: pre-eclampsia, placenta previa, placental abruption, cesarean section) and adverse pregnancy outcomes (at least one of the following: PTB, LBW, SGA, low Apgar score at 5 min, congenital anomalies, admission to a NICU) were used. Crude and adjusted RRs with 95% confidence intervals (CI) were calculated. Maternal socio-demographic characteristics were considered as potential confounders. Models were adjusted for maternal age (25–29 years as reference), study period (2005–2009 vs. 2010–2014), education (secondary or less vs. higher), ethnicity (non-Estonian vs. Estonian) and marital status (single vs. married or cohabiting). Models for LBW and VLBW as outcome were additionally adjusted for the infant’s sex. Linear regression was used to quantify differences in mean birthweight and gestational age between the IVF and SC cohorts. Models were adjusted as described above. Modified Poisson regression models with a combined category of the pregnancy-related complications or the adverse pregnancy outcomes as the dependent variables, and maternal age, education, ethnicity, marital status and study period as the predictor variables, were applied to the IVF and SC cohorts separately.

Statistical analyses were performed using Stata 14 (StataCorp LP, College Station, TX, USA).

## Results

We compared data on liveborn singletons to primiparas after IVF (1778 newborns) and SC pregnancies (33,555 newborns) (Table 1). As expected, IVF mothers were older than SC mothers, with a mean age of 32.5 ± 3.8 and 28.6 ± 3.3 years, respectively. The proportion of IVF births among married mothers was two-fold that of cohabiting mothers. No marked differences were seen in the proportion of IVF births by maternal educational level or ethnicity. IVF singletons had a slightly shorter mean gestational age than SC singletons (38.9 ± 2.2 and 39.3 ± 1.8 weeks, respectively) and a lower mean birthweight (3400 ± 609 and 3473 ± 532 g, respectively). The linear regression models adjusted for maternal age, education, ethnicity, marital status and study period (additionally, for infant’s sex for birthweight) demonstrated 0.3 weeks (95% CI 0.3–0.4 weeks) shorter gestational age and 48 g (95% CI 22–74 g) lower birthweight in the IVF cohort. The proportion of adverse conditions was higher in the IVF cohort for any of the available pregnancy-related outcomes (Table 1).

**Table 1:** Characteristics of births after IVF and SC pregnancies^a^

Characteristic	IVF	SC	Proportion of
	*n* (%)	*n* (%)	IVF births (%)
Total	1778	33,555	5.0
Year of birth			
2005–2009	708 (39.8)	16,980 (50.6)	4.0
2010–2014	1070 (60.2)	16,575 (49.4)	6.1
*Maternal socio-demographic background*			
Age (years)			
25–29	436 (24.5)	22,849 (68.1)	1.9
30–34	778 (43.8)	8342 (24.9)	8.5
35–40	564 (31.7)	2364 (7.0)	19.3
Ethnicity			
Estonian	1248 (70.2)	23,851 (71.1)	5.0
Russian	486 (27.3)	8775 (26.2)	5.2
Other	44 (2.5)	929 (2.8)	4.5
Education			
Higher	1010 (56.8)	19,519 (58.2)	4.9
Secondary	716 (40.3)	13,040 (38.9)	5.2
Basic	52 (2.9)	996 (3.0)	5.0
Marital status			
Married	935 (52.6)	11,779 (35.1)	7.4
Cohabiting	743 (41.8)	19,698 (58.7)	3.6
Single	100 (5.6)	2078 (6.2)	4.6
*Pregnancy-related characteristics*			
Pre-eclampsia			
Yes	78 (4.4)	1007 (3.0)	7.2
No/ unknown	1700 (95.6)	32,548 (97.0)	5.0
Placenta previa			
Yes	22 (1.2)	51 (0.2)	30.1
No/ unknown	1756 (98.8)	33,504 (99.8)	5.0
Placental abruption			
Yes	32 (1.8)	250 (0.7)	11.3
No/ unknown	1746 (98.2)	33,305 (99.3)	5.0
Mode of delivery			
Vaginal	994 (55.9)	22,920 (68.3)	4.2
Instrumental	164 (9.2)	2920 (8.7)	5.3
Planned cesarean section	217 (12.2)	1698 (5.1)	11.3
Emergency cesarean section	403 (22.7)	6017 (17.9)	6.3
*Pregnancy outcome*			
Infant’s sex			
Male	893 (50.2)	17,200 (51.3)	4.9
Female	885 (49.8)	16,355 (48.7)	5.1
Gestational age (weeks)			
< 32	30 (1.7)	270 (0.8)	10.0
32–36	123 (6.9)	1398 (4.2)	8.1
≥ 37	1625 (91.4)	31,887 (95.0)	4.8
Birthweight (g)			
< 1500	22 (1.2)	219 (0.7)	9.1
1500–2499	87 (4.9)	927 (2.8)	8.6
2500–3499	872 (49.0)	15,792 (47.1)	5.2
3500–4499	764 (43.0)	15,962 (47.6)	4.6
≥ 4500	33 (1.9)	655 (2.0)	4.8
Small for gestational age			
Yes	46 (2.6)	613 (1.8)	7.0
No	1732 (97.4)	32,942 (98.2)	5.0
Apgar score at 5 min			
< 7	30 (1.7)	401 (1.2)	7.0
≥ 7	1747 (98.3)	33,116 (98.7)	5.0
Unknown	1 (0.1)	38 (0.1)	2.6
Congenital anomalies			
Yes	40 (2.3)	506 (1.5)	7.3
No/ unknown	1738 (97.8)	33,049 (98.5)	5.0
Admission to a NICU			
Yes	300 (16.9)	4369 (13.0)	6.4
No/ unknown	1478 (83.1)	29,186 (87.0)	4.8

^a^Liveborn singletons to primiparas in Estonia, 2005–2014

*IVF* In vitro fertilization, *NICU* Neonatal intensive care unit, *SC* Spontaneously conceived

Crude Poisson regression analysis revealed a significantly higher risk of PTB (both, spontaneous and iatrogenic PTB), VPTB, LBW, VLBW, SGA, congenital anomalies and admission to a NICU among the IVF singletons compared with their SC counterparts. The risk of low Apgar score (< 7) had somewhat increased, but not statistically significant point estimate. IVF mothers were at a higher risk of pre-eclampsia, placental complications and cesarean section (Table 2).

**Table 2:** Risk of selected complications and adverse outcomes after IVF vs. SC pregnancies^a^

Complication/ outcome	IVF	SC	Crude RR (95% CI)	RR (95% CI) adjusted for:		
				Maternal age	+ Study period	+ Ethnicity, education, marital status
*Pregnancy-related complications* ^b^	650	8206	1.49 (1.40–1.59)	1.28 (1.19–1.36)	1.28 (1.20–1.37)	1.28 (1.19–1.36)
Pre-eclampsia	78	1007	1.46 (1.17–1.83)	1.24 (0.97–1.57)	1.25 (0.98–1.59)	1.25 (0.98–1.59)
Placental complications^c^	49	299	3.09 (2.30–4.17)	2.71 (1.96–3.76)	2.74 (1.98–3.79)	2.74 (1.97–3.79)
Placenta previa	22	51	8.14 (4.95–13.39)	7.51 (4.27–13.23)	7.57 (4.29–13.35)	7.15 (4.04–12.66)
Placental abruption	32	250	2.42 (1.68–3.48)	2.08 (1.40–3.07)	2.10 (1.42–3.10)	2.12 (1.43–3.14)
Cesarean section^d^	620	7715	1.52 (1.42–1.62)	1.28 (1.20–1.37)	1.29 (1.20–1.38)	1.28 (1.20–1.37)
Planned	217	1698	2.41 (2.11–2.75)	1.89 (1.64–2.17)	1.92 (1.67–2.21)	1.92 (1.67–2.21)
Emergency	403	6017	1.26 (1.16–1.38)	1.09 (1.00–1.20)	1.10 (1.00–1.20)	1.09 (0.99–1.19)
*Adverse pregnancy outcomes* ^e^	400	5720	1.32 (1.21–1.44)	1.18 (1.07–1.29)	1.18 (1.07–1.29)	1.17 (1.07–1.29)
Preterm birth (< 37 weeks)	153	1668	1.73 (1.48–2.01)	1.49 (1.26–1.76)	1.49 (1.26–1.76)	1.51 (1.28–1.78)
Spontaneous preterm birth	48	616	1.47 (1.10–1.96)	1.30 (0.95–1.77)	1.30 (0.95–1.77)	1.32 (0.97–1.80)
Iatrogenic preterm birth	105	1052	1.88 (1.55–2.29)	1.60 (1.31–1.96)	1.61 (1.31–1.97)	1.62 (1.32–1.98)
Very preterm birth (< 32 weeks)	30	300	2.10 (1.44–3.05)	1.47 (0.99–2.18)	1.48 (0.99–2.20)	1.49 (1.00–2.23)
Low birthweight (< 2500 g)	109	1146	1.80 (1.48–2.17)	1.46 (1.19–1.78)^f^	1.46 (1.20–1.78)^f^	1.47 (1.20–1.80)^f^
Very low birthweight (< 1500 g)	22	241	1.90 (1.23–2.93)	1.32 (0.84–2.06)^f^	1.31 (0.84–2.05)^f^	1.31 (0.84–2.06)^f^
Small for gestational age	46	613	1.42 (1.05–1.90)	1.23 (0.91–1.67)	1.23 (0.91–1.67)	1.23 (0.91–1.66)
Apgar score at 5 min < 7	30	401	1.41 (0.98–2.04)	1.20 (0.81–1.77)	1.22 (0.82–1.80)	1.20 (0.81–1.77)
Congenital anomalies	40	506	1.49 (1.09–2.05)	1.50 (1.08–2.09)	1.52 (1.09–2.12)	1.51 (1.08–2.11)
Admission to a NICU	300	4369	1.30 (1.16–1.44)	1.14 (1.02–1.27)	1.13 (1.02–1.27)	1.13 (1.01–1.26)

^a^Liveborn singletons to primiparas in Estonia, 2005–2014

^b^At least one of the following complications: pre-eclampsia, placenta previa, placental abruption, cesarean section

^c^Placenta previa or placental abruption

^d^Planned or emergency cesarean section

^e^At least one of the following outcomes: preterm birth, low birthweight, small for gestational age, Apgar score at 5 min < 7, congenital anomalies, admission to a NICU

^f^Additionally adjusted for infant’s sex

*CI* Confidence interval, *IVF* In vitro fertilization, *NICU* Neonatal intensive care unit, *RR* Relative risk (prevalence proportion ratio), *SC* spontaneously conceived

Adjustment for maternal age markedly attenuated the associations between IVF and adverse outcomes, and the risks for spontaneous PTB, VPTB, VLBW, SGA and pre-eclampsia lost their statistical significance. Further adjustments for study period, maternal education, ethnicity and marital status barely altered the risk estimates. The highest risks were seen for placenta previa (RR 7.15; 95% CI 4.04–12.66), placental abruption (RR 2.12; 95% CI 1.43–3.14) and planned cesarean section (RR 1.92; 95% CI 1.67–2.21). The risks remained stable in the two study periods, with the exception of decreasing rates of planned cesarean section (RR 0.84; 95% CI 0.77–0.92) and congenital anomalies (RR 0.84; 95% CI 0.71–0.99). The risk of having at least one of the pregnancy-related complications (RR 1.28; 95% CI 1.19–1.36) or at least one of the adverse pregnancy outcomes (RR 1.17; 95% CI 1.07–1.29) was significantly higher in the IVF cohort (Table 2).

Additional analysis separately in the IVF and SC cohorts demonstrated an increased risk of at least one of the pregnancy-related complications or at least one of the adverse pregnancy outcomes among less educated, non-Estonian or single mothers; that risk grew with maternal age. No marked difference in the magnitudes of point estimates of the RRs for maternal age, education, ethnicity or marital status in the two cohorts was evident, but 95% confidence intervals were wider for the IVF cohort because of the small number of births (Table 3).

**Table 3:** Risk of complications and adverse outcomes after IVF and SC pregnancies^a^ by maternal socio-demographic background

Characteristic	Adjusted RR (95% CI)^b^	
	IVF	SC
*Pregnancy-related complications* ^c^		
Age (30–34 vs. 25–29 years)	1.30 (1.09–1.56)	1.28 (1.23–1.33)
Age (35–40 vs. 25–29 years)	1.55 (1.30–1.86)	1.57 (1.48–1.67)
Education (secondary or less vs. higher)	1.29 (1.14–1.46)	1.18 (1.14–1.23)
Ethnicity (non-Estonian vs. Estonian)	1.02 (0.89–1.16)	1.09 (1.05–1.14)
Marital status (single vs. married)	1.18 (0.94–1.48)	1.09 (1.02–1.17)
Period (2005–2009 vs. 2010–2014)	1.13 (1.00–1.28)	1.04 (1.00–1.08)
*Adverse pregnancy outcomes* ^d^		
Age (30–34 vs. 25–29 years)	1.09 (0.86–1.37)	1.15 (1.08–1.21)
Age (35–40 vs. 25–29 years)	1.31 (1.03–1.66)	1.42 (1.31–1.54)
Education (secondary or less vs. higher)	1.11 (0.93–1.33)	1.16 (1.11–1.22)
Ethnicity (non-Estonian vs. Estonian)	1.15 (0.96–1.38)	1.16 (1.10–1.22)
Marital status (single vs. married)	1.12 (0.80–1.58)	1.19 (1.09–1.30)
Period (2005–2009 vs. 2010–2014)	0.89 (0.74–1.06)	1.00 (0.96–1.05)

^a^Liveborn singletons to primiparas in Estonia, 2005–2014

^b^Mutually adjusted for all other socio-demographic characteristics in the table

^c^At least one of the following complications: pre-eclampsia, placenta previa, placental abruption, cesarean section

^d^At least one of the following outcomes: preterm birth (< 37 weeks), low birthweight (< 2500 g), small for gestational age, Apgar score at 5 min < 7, congenital anomalies, admission to a neonatal intensive care unit

*CI* Confidence interval, *IVF* In vitro fertilization, *RR* Relative risk (prevalence proportion ratio), *SC* Spontaneously conceived

## Discussion

The findings of our study revealed that singletons born after IVF were slightly disadvantaged at birth. Their mean gestational age was shorter, and their mean birthweight was lower than that of the SC singletons. The IVF singletons had a higher risk of congenital anomalies, and treatment in a NICU was more prevalent among them. A significantly elevated risk of a low Apgar score among the IVF singletons was not apparent. IVF pregnancies carried a significantly increased risk of placental complications and cesarean section; the risk of pre-eclampsia was borderline. Our study period was too short to identify improvements in the IVF pregnancy outcomes.

The observed risk of PTB and LBW was similar to that found in the first descriptive study of the IVF newborns in Estonia that analyzed the period of 2001–2009 without prior validation of the IVF status in the birth records [27]. The current risk estimates for PTB, VPTB, LBW and VLBW were in line with the majority of the previous studies that compared IVF singletons with SC singletons [5, 18, 19], and the magnitudes were somewhat lower than those reported in a recent meta-analysis (PTB 1.51 vs. 1.71 and LBW 1.47 vs. 1.61) [11]. It has been concluded that the more recent the study cohort, the lower the risk of PTB, VPTB, LBW and VLBW [10, 28]. The authors have expressed the opinion that decreasing time trend in these risks is caused by general improvement of clinical and laboratory skills, wider use of frozen embryos, milder ovarian stimulation, healthier lifestyle in couples seeking fertility treatment and more careful selection of patients and embryos over time. A few papers have shown no difference in PTB and LBW between IVF and SC singletons after single embryo transfer [14]. Fujii et al. [29] demonstrated no significant risk of LBW after adjustment for gestational age.

In line with the publications on the need for investigating spontaneous and iatrogenic PTB separately [30, 31], we found that risk of both types of PTB was elevated among IVF pregnancies compared with those conceived spontaneously. A statistically significant higher RR occurred for iatrogenic PTB, but in the case of longer study period the risk of spontaneous PTB would have also become significant.

In the current study, increased risk of SGA in the IVF cohort did not emerge, indicating that shorter gestational age rather than fetal growth problems contributed to the lower birthweight. Evidence on the risk of SGA among the IVF singletons varies from lower [21] or similar [14, 32] to the increased risk [28] in comparison with the SC singletons. However, the trend towards equal proportion of SGA singletons in the more recent IVF and SC cohorts has been shown [10, 28].

It has been confirmed that IVF pregnancies are associated with an increased prevalence of cesarean section deliveries [9, 11], but only in few studies were planned and emergency cesarean sections analyzed separately [32, 33]. Although the risk of pooled cesarean sections in the current study (28% increase) was lower than that reported in the meta-analyses (56–58% increase) [9, 11], the risk of planned cesarean section in the IVF cohort was nearly double the risk in the SC cohort. The significantly increased risk of placenta previa, which is known to be an important indication for planned cesarean section [34] and strongly related to the IVF pregnancies [11, 21, 35], likely contributed to the elevated risk of planned cesarean section in the IVF cohort in the current study. However, the rare occurrence of placenta previa could not be responsible for such increase. Thus, the high proportion of planned cesarean sections might be explained by other pregnancy complications, and may even be more associated with anxiety about the delivery and the choice to give birth by cesarean section [10, 32, 33].

The 51% increased risk of congenital anomalies observed in the IVF cohort were in accord with previous research. Depending on different definitions, follow-up time and selection of the comparison group, risk estimates from 1.36 [36] to 1.67 [9] have been published in meta-analyses for IVF singletons. The highest risk of congenital anomalies (6.07) has recently been reported in the study by Qin et al. [19] in which pregnancy outcome in the IVF group was compared with that in the fertile group, while subfertile mothers were excluded. Because the IVF singletons were at a higher risk of adverse conditions, they required intensive medical attention more frequently and were admitted to the NICUs. In our study, the nature and duration of the treatment was unknown.

It is well known that pregnancy-related complications and adverse pregnancy outcomes are associated with maternal health behaviors (tobacco smoking, alcohol consumption, obesity) that are partly determined by the socio-demographic characteristics [37–39]. The same lifestyle factors are related to infertility [40, 41]. Since our study relied exclusively on the EMBR data, we could not use information on maternal health behaviors. Although the EMBR collects information about smoking in pregnancy, these data are incomplete and with poor validity [25, 42]. Thus, we included maternal education, ethnicity and marital status as proxies for health behavior in the regression models to control for confounding. According to the Estonian health behavior survey 2014 data, daily smoking, hazardous drinking and obesity in women aged 25–44 years were more prevalent among less educated and unmarried respondents. A clear difference between ethnic groups was evident only for smoking – daily smoking was more common among non-Estonians (mainly Russians) [43].

Comparing the results from the multiple Poisson regression models for each adverse condition, it appeared that adjustment for maternal age reduced the RRs considerably, indicating that age was a strong confounder of the association between IVF and outcome. Further adjustments for maternal education, ethnicity, marital status and study period had virtually no impact on the risk estimates. This result is consistent to that found by Räisänen et al. [21] where models were additionally adjusted for socio-economic status defined by maternal occupation. As previously observed in Estonia [22], maternal education, ethnicity and marital status were independently associated with the perinatal outcome. We confirmed the effect of maternal socio-demographic characteristics on pooled pregnancy-related complications and adverse pregnancy outcomes separately in the IVF and SC cohorts, with magnitudes quite close to each other. Younger age, higher education, Estonian ethnicity and being married were beneficial factors in both cohorts.

The average maternal age at first birth rose from 25 to 27 years in Estonia during 2005–2014 [44]. It is well established that advanced age is associated with an increased risk of pregnancy-related complications and adverse pregnancy outcomes [45, 46], but the cut-off ages for the different outcomes can be different and can even be under the age of 35 years [47]. Postponement of pregnancies reduces the chances of a spontaneous pregnancy and therefore increases the need for infertility treatment [48]. In Estonia, all women up to 40 years of age have equal access to the IVF treatment. The fact that an unlimited number of cycles is covered by the EHIF explains why differences in the prevalence of the IVF pregnancies by maternal education (as a marker of socio-economic status) were not seen in our study. The IVF treatment can remain financially out of reach only for women above the age of 40 years.

The major strength of our study is that it analyzes a relatively large dataset from the EMBR database with prior assessment of the validity of the IVF status registration. Using the whole cohort of liveborn singletons to primiparas in Estonia from 2005 to 2014 increased the study power. The EMBR provided a good opportunity to measure the risk of pregnancy-related complications and adverse pregnancy outcomes after IVF in relation to the maternal socio-demographic background. However, relying only on the birth registry data also has some weaknesses. The EMBR is not designed to contain neither details of the IVF treatment (diagnostic and treatment modalities, number of treatment cycles), causes of infertility, nor the complex of lifestyle factors that can influence reproductive health. For these reasons, more efficient control of confounding was not possible to do. Similarly, the EMBR database does not include data on specific congenital anomalies and some interesting topics like increased risk of congenital heart defects in IVF**-**conceived children [49] left untouched. The congenital malformations in detail are worth to be researched in Estonia when the birth registry gets more mature and the cumulative number of IVF births is larger than now.

Since tick boxes are used on the birth notification form for the pregnancy-related complications, “no” and “unknown” answers cannot be separately identified. This possible underreporting of some outcomes is likely non-differential that reduces observed risk estimates. In addition, we were not able to exclude pregnancies after ovulation induction or intrauterine insemination from the comparison SC cohort, which could slightly underestimate the associations [11].

## Conclusions

The increased risk of pregnancy-related complications and adverse pregnancy outcomes was observed in the Estonian cohort of IVF singletons in comparison with the cohort of SC singletons. The relative risk estimates grew with maternal age, but were not influenced by the maternal education, ethnicity and marital status. To monitor the efficacy and safety of the used assisted reproductive technology, a specialized country-wide register should be created in Estonia.

## Abbreviations

CI: Confidence interval
EHIF: Estonian Health Insurance Fund
EMBR: Estonian Medical Birth Registry
IVF: In vitro fertilization
LBW: Low birthweight
NICU: Neonatal intensive care unit
PTB: Preterm birth
RR: Relative risk (prevalence proportion ratio)
SC: Spontaneously conceived
SGA: Small for gestational age
VLBW: Very low birthweight
VPTB: Very preterm birth
